# Genetic Analysis Reveals an Unexpected Role of BMP7 in Initiation of Ureteric Bud Outgrowth in Mouse Embryos

**DOI:** 10.1371/journal.pone.0019370

**Published:** 2011-04-28

**Authors:** Alexandre Gonçalves, Rolf Zeller

**Affiliations:** Developmental Genetics, Department of Biomedicine, University of Basel Medical Faculty, Basel, Switzerland; Laboratoire Arago, France

## Abstract

**Background:**

Genetic analysis in the mouse revealed that GREMLIN1 (GREM1)-mediated antagonism of BMP4 is essential for ureteric epithelial branching as the disruption of ureteric bud outgrowth and renal agenesis in *Grem1*-deficient embryos is restored by additional inactivation of one *Bmp4* allele. Another BMP ligand, BMP7, was shown to control the proliferative expansion of nephrogenic progenitors and its requirement for nephrogenesis can be genetically substituted by *Bmp4*. Therefore, we investigated whether BMP7 in turn also participates in inhibiting ureteric bud outgrowth during the initiation of metanephric kidney development.

**Methodology/Principal Findings:**

Genetic inactivation of one *Bmp7* allele in *Grem1*-deficient mouse embryos does not alleviate the bilateral renal agenesis, while complete inactivation of *Bmp7* restores ureteric bud outgrowth and branching. In mouse embryos lacking both *Grem1* and *Bmp7*, GDNF/WNT11 feedback signaling and the expression of the *Etv4* target gene, which regulates formation of the invading ureteric bud tip, are restored. In contrast to the restoration of ureteric bud outgrowth and branching, nephrogenesis remains aberrant as revealed by the premature loss of *Six2* expressing nephrogenic progenitor cells. Therefore, very few nephrons develop in kidneys lacking both *Grem1* and *Bmp7* and the resulting dysplastic phenotype is indistinguishable from the one of *Bmp7*-deficient mouse embryos.

**Conclusions/Significance:**

Our study reveals an unexpected inhibitory role of BMP7 during the onset of ureteric bud outgrowth. As BMP4, BMP7 and GREM1 are expressed in distinct mesenchymal and epithelial domains, the localized antagonistic interactions of GREM1 with BMPs could restrict and guide ureteric bud outgrowth and branching. The robustness and likely significant redundancy of the underlying signaling system is evidenced by the fact that global reduction of *Bmp4* or inactivation of *Bmp7* are both able to restore ureteric bud outgrowth and epithelial branching in *Grem1*-deficient mouse embryos.

## Introduction

Development of the metanephric kidney depends on reciprocal signaling interactions between ureteric epithelium and the surrounding metanephric mesenchyme. Metanephric kidney morphogenesis is initiated by formation of one ureteric bud in the caudal Wolffian duct in proximity to the metanephric mesenchyme [1], [2]. Rearrangement of the Wolffian duct epithelial cells results in the incorporation of the cells with highest activity of the GDNF receptor RET into the nascent ureteric bud [3]. The site of ureteric bud formation is restricted to its proper location by complex molecular interactions as supernumerary ureteric buds can be induced in a number of experimental and genetic conditions. For example, exposure of urogenital ridges in culture to an excess of GNDF ligand [4] or the BMP antagonist GREMLIN1 (GREM1, see below) [5] induces formation of supernumerary ureteric buds and branches. Additional ureteric buds form in mouse embryos lacking either *Slit2* or *Robo2*, which are normally required to restrict *Gdnf* expression to caudal mesenchyme [6]. Ectopic ureteric buds are also apparent in *Sprouty1* (*Spry1*)-deficient mouse embryos and molecular analysis revealed that SPRY1 reduces the sensitivity of the Wolffian duct to GDNF [7]. However, complete inactivation of *Gdnf* or *Ret* in combination with *Spry1* results in formation of only one ureteric bud [8]. This is due to the activity of FGF10, as additional inactivation of *Fgf10* in mouse embryos lacking both *Gdnf* and *Spry1* completely abolishes ureteric bud formation. These and other studies reveal that formation of the ureteric bud is controlled by an at least partially redundant signaling system. The transcriptional regulators *Etv4* and *Etv5* act downstream of GDNF/RET and FGF10 signaling to control formation of the ureteric bud tip domain [8], [9], [10], [11]. Subsequently, the ureteric bud invades the metanephric mesenchyme and mesenchymal GDNF/RET signaling controls branching of the ureteric epithelial tree, as revealed by extensive genetic analysis in the mouse [1], [2]. During initiation of outgrowth, *Wnt11* expression is activated in the ureteric epithelial tips and WNT11 propagates mesenchymal *Gdnf* expression as part of an auto-regulatory epithelial-mesenchymal (e-m) feedback signaling loop [12]. As branching morphogenesis proceeds, the ureteric tips secrete WNT9b, which induces nephrogenesis [13]. The nephrogenic progenitors express the transcription factor *Six2*, which is required for their self-renewal [14], [15].

Several BMP ligands and their receptors are expressed during metanephric kidney organogenesis. In particular, *Bmp7* is expressed by both the ureteric epithelium and metanephric mesenchyme [16], [17]. Kidneys of *Bmp7*-deficient mouse embryos are hypodysplastic as mesenchymal BMP7 is essential to maintain the nephrogenic progenitors during kidney development [18], [19]. Genetic evidence for potential functional compensation was obtained by expressing *Bmp4* under control of the *Bmp7* locus in *Bmp7* ^Δ/Δ^ embryos, which restores metanephric kidney development [20]. Some mouse embryos heterozygous for a *Bmp4* loss-of-function mutation display defects in ureteric stalk elongation [21]. In contrast to *Bmp7*, *Bmp4* is expressed predominantly by the tailbud-derived mesenchyme that envelops the cloaca and caudal Wolffian duct and promotes segmentation of the ureteric epithelium into the ureter and collecting duct system [22]. Treatment of metanephric kidney primordia with recombinant BMP4 interferes with epithelial branching and induces differentiation of collecting ducts into epithelia with urethral phenotypes, which together with other results indicated that BMP4 activity changes dynamically during kidney development [21], [22], [23]. One key modulator of BMP activity in mouse embryos is the extra-cellular antagonist GREM1 [17], [24]. We previously established that *Grem1*, which is expressed by the mesenchyme surrounding the ureteric bud, is required to initiate its outgrowth [17]. The bilateral renal agenesis in *Grem1*-deficient mice is restored by additional inactivation of one *Bmp4* allele [5]. This restoration indicated that GREM1 antagonizes BMP4 in the mesenchyme surrounding the nascent ureteric bud, which enables its outgrowth and invasion of the metanephric mesenchyme.

We hypothesized that GREM1 could antagonize BMP7 in addition to BMP4 during kidney organogenesis, as is the case during limb bud development [24]. Analysis of newborn mice lacking both *Grem1* and *Bmp7* revealed the presence of two distinct but hypodysplastic kidneys. Molecular analysis established that ureteric bud outgrowth and branching, GNDF/WNT11-mediated epithelial-mesenchymal feedback signaling and initiation of nephrogenesis were restored in *Grem1* ^Δ/Δ^, *Bmp7* ^Δ/Δ^ embryos. In contrast, BMP7-dependent maintenance of *Six2* positive nephrogenic progenitors was not restored, which caused the hypodysplastic kidney phenotype. Taken together, our studies reveal that GREM1-mediated antagonism of BMP4 and BMP7 during initiation of metanephric kidney development reduces overall BMP activity such that ureteric bud outgrowth and branching are initiated.

## Results

**Figure 1:**
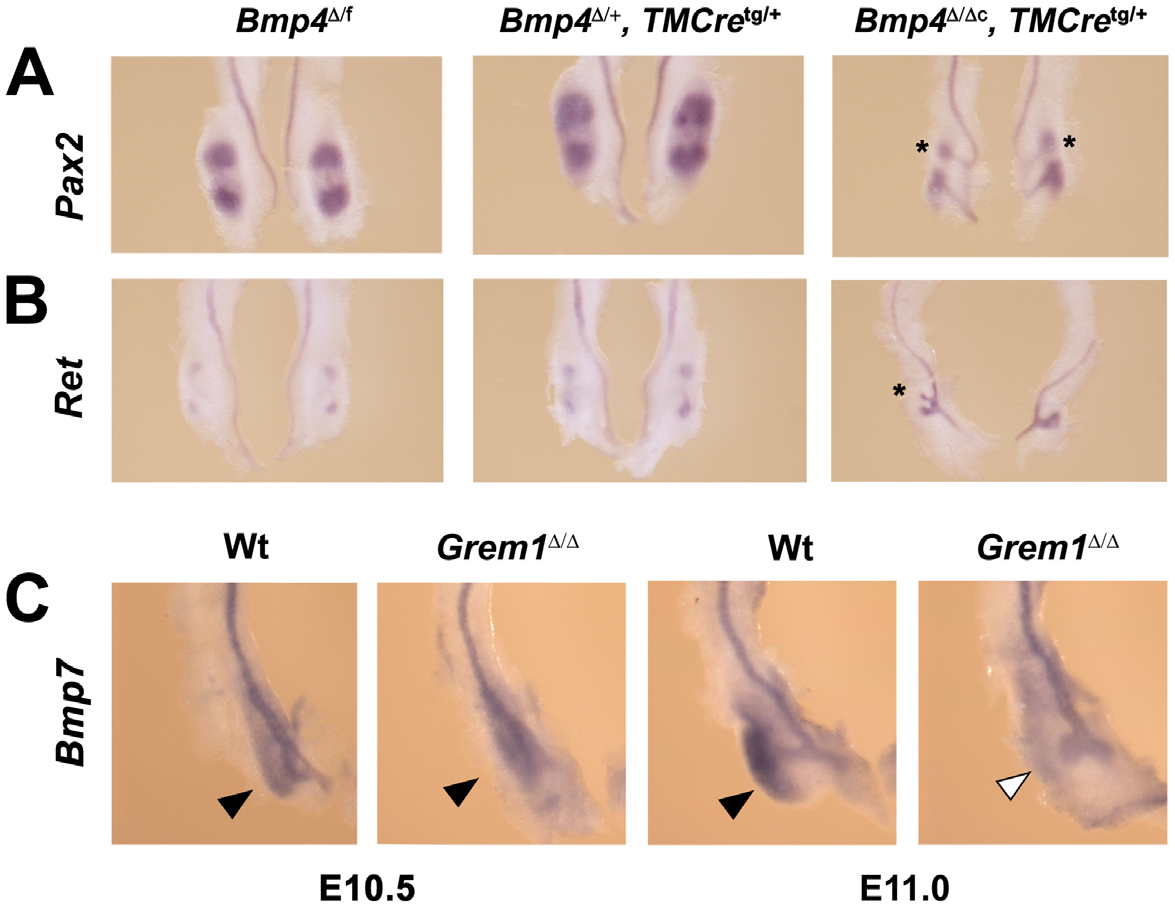
*Bmp4* and *Bmp7* during initiation of ureteric bud outgrowth. (A, B) Kidney rudiments were isolated at embryonic days E11.5 from pregnant females injected with tamoxifen at E8.75–E9.5 of gestation, which inactivates the remaining conditional *Bmp4* allele within 24 hours following injection [24], i.e. prior to initiation of ureteric bud outgrowth (*Bmp4* ^Δ/Δc^, *TMCre* ^tg/+^). The genotypes are indicated above the panels and *Bmp4* ^Δ/f^ and *Bmp4* ^Δ/+^, *TMCre* ^tg/+^ embryos are shown as wild-type controls. Note that all embryos (incl. controls) received tamoxifen. *Pax2* expression (A) marks metanephric mesenchyme around the ureteric bud epithelia and the epithelia itself, while *Ret* expression (B) marks the tips of the invading ureteric buds. Asterisks mark the ectopic epithelial buds observed in 50% of all *Bmp4* ^Δ/Δc^, *TMCre* ^tg/+^ kidney rudiments at E11.5 (n = 5/10). Note the developmental delay of kidney development in *Bmp4* ^Δ/Δc^, *TMCre* ^tg/+^ embryos and the ectopic expression of *Ret* and *Pax2*. Ectopic buds were never observed in metanephric kidney rudiments isolated from *Bmp4* ^Δ/f^ and *Bmp4* ^Δ/*+*^, *TMCre* ^tg/+^ embryos. (C) In *Grem1*-deficient (*Grem1* ^Δ/Δ^) metanephric kidney rudiments, *Bmp7* expression (black arrowheads) appeared initially normal (E10.5), but was rapidly lost from the metanephric mesenchyme (white arrowhead; E11.0) while expression remained in the developmentally arrested epithelium.

In an attempt to gain further mechanistic insights into the inhibitory role of BMPs in the formation of the ureteric bud and initiation of its outgrowth, *Bmp4* was genetically inactivated in *Bmp4* ^Δ/f^ mouse embryos carrying a tamoxifen-inducible Cre recombinase transgene (*Bmp4* ^Δ/Δc^, *TMCre* ^tg/+^, Figure 1A, 1B) by injecting pregnant females with tamoxifen between embryonic day E8.75–E9.5. This will inactivate the remaining hypomorphic *Bmp4* allele within less than 24 hours [24], i.e. before the onset of ureteric bud outgrowth around E10.75–11.0. Embryos were isolated ≥48 hours later at E11.5 and genotyping revealed approximately 50% lethality of the *Bmp4* ^Δ/Δc^, *TMCre* ^tg/+^ embryos. This is likely due to vital *Bmp4* functions in other embryonic tissues as the overall lethality of all embryos having received tamoxifen was 23% (data not shown). Molecular analysis of the surviving *Bmp4*-deficient embryos (*Bmp4* ^Δ/Δc^, *TMCre* ^tg/+^) at E11.5 revealed a consistent and general developmental delay, which is apparent when comparing the extent of ureteric bud outgrowth and branching with age-matched littermate controls (also having received tamoxifen; Figure 1A, 1B). One ectopic epithelial bud (asterisks in Figure 1A, 1B) was observed in close proximity to the endogenous ureteric bud in half of all *Bmp4*-deficient kidneys analyzed (Figure 1A, 1B; n = 5/10). Such ectopic buds were not detected in the *Bmp4* ^Δ/f^ and *Bmp4* ^Δ/+^, *TMCre* ^tg/+^ kidney rudiments and other wild-type controls (Figure 1A, 1B; n>10). The formation of maximally one ectopic ureteric bud per *Bmp4*-deficient kidney rudiment contrasted sharply with the several ectopic buds observed in both wild-type and *Grem1*-deficient kidneys treated with recombinant GREM1 protein *in vitro* [5].

**Figure 2:**
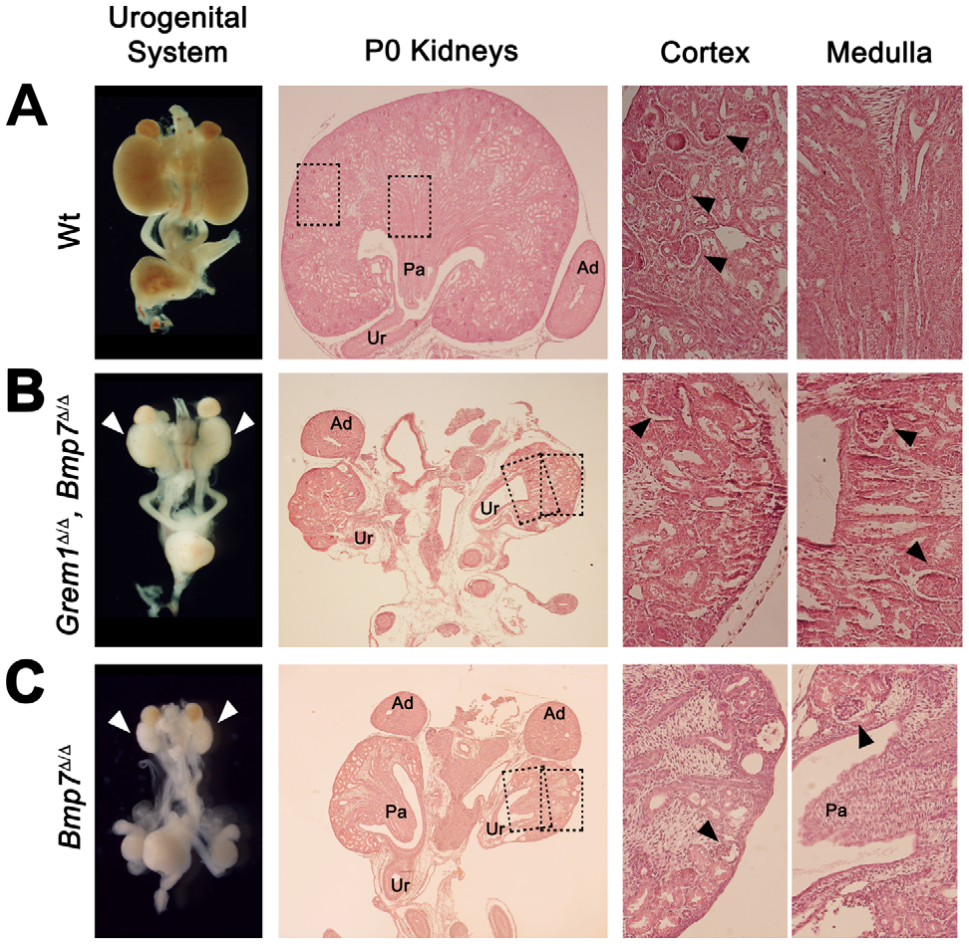
Additional genetic inactivation of *Bmp7* in *Grem1*-deficient mouse embryos only partially restores metanephric kidney development. Morphological analysis of kidneys of newborn mice (postnatal day P0) revealed the presence of two hypodysplastic kidneys in most cases in *Grem1* ^Δ/Δ^, *Bmp7* ^Δ/Δ^ mice (see also Table 1). (A) Wild-type (+/+) urogenital system at birth (left panel). Histological analysis revealed the morphology of the ureter, an organized collecting duct system in the medulla and many glomeruli in the cortex (only some are indicated by black arrowheads). (B) In contrast, two small, hypodysplastic kidneys (white arrowheads) formed in *Grem1* ^Δ/Δ^, *Bmp7* ^Δ/Δ^ mice (left panel). Histological analysis revealed the drastic reduction and disorganization of both cortex and medulla, while the ureter, indicative of ureteric epithelial branching was present. Black arrowheads point to the few glomeruli that formed, some of them located within the rudimentary medulla. (C) The hypodysplastic kidney phenotype of *Bmp7*-deficient mice (white arrowheads, left panel) [18], [19]. Note the similarity of the kidney phenotypes of *Bmp7* ^Δ/Δ^ and *Grem1* ^Δ/Δ^, *Bmp7* ^Δ/Δ^ mice at both gross-morphological and histological levels. White arrowheads: hypodysplastic kidneys; Ad: adrenal glands; Pa: papilla; Ur: ureter; stippled boxes: enlargements shown in the right panels; black arrowheads: glomeruli.

Our genetic analysis of GREM1-mediated BMP antagonism during limb bud development established that while its antagonistic interaction with BMP4 is functionally most relevant, *Grem1* also interacts with *Bmp7* [24]. During kidney development, *Bmp7* is expressed both by the epithelium and mesenchyme surrounding the ureteric bud tip (Figure 1C) [16], [17]. *Bmp7* expression remained normal in *Grem1*-deficient mouse embryos at E10.5 (black arrowheads, Figure 1C), but was lost from the metanephric mesenchyme by E11.0 (white arrowhead in Figure 1C). This analysis suggested that GREM1 could potentially antagonize BMP7 during initiation of metanephric kidney development and that mesenchymal *Bmp7* expression depends on GREM1 similar to the auto-regulatory feedback interactions of GREM1 and BMP4 during early limb bud development [24]. Next, we generated mice lacking both *Grem1* and *Bmp7* to determine to what extent metanephric kidney development was restored at birth. As expected, bilateral renal agenesis was observed in about 90% of all *Grem1*-deficient mice at birth in the presence of either one or two functional *Bmp7* alleles (Table 1) [5], [17]. In contrast, two small kidneys formed in the vast majority of mice lacking both *Grem1* and *Bmp7* (Table 1 and Figure 2). Gross-morphological and histological analysis revealed the presence of ureters and collecting duct systems, but in contrast to the normal morphology of wild-type (Figure 2A) and *Grem1* ^Δ/Δ^, *Bmp4* ^Δ/+^ kidneys (Figure S1) [5], the kidneys of *Grem1* ^Δ/Δ^, *Bmp7* ^Δ/Δ^ newborn mice were severely hypodysplastic (Table 1 and Figure 2B). In fact, they were phenotypically identical to kidneys of *Bmp7*-deficient mice (Table 1 and Figure 2C). The thickness of both the cortex and medulla were drastically reduced and only a small and variable number of glomeruli had formed in *Grem1* ^Δ/Δ^, *Bmp7* ^Δ/Δ^ and *Bmp7*-deficient kidneys (arrowheads in Figure 2B, 2C and Figure S1). In contrast to *Bmp4*-deficient kidney rudiments (Figure 1A, 1B), no ectopic ureteric buds were observed in mouse embryos lacking *Bmp7* (data not shown).

**Table 1:** Prevalence of the complete renal agenesis in *Bmp7* and *Grem1*-deficient and compound mutant mice at birth.

Genotypes	2 kidneys	1 kidney	0 kidneys	total
***Grem1*** ^Δ/Δ^	**0**	**1 (10%)**	**9 (90%)**	**10**
*Grem1* ^Δ/Δ^; *Bmp7* ^Δ/+^	1 (5%)*	2 (10%)*	17 (85%)	20
***Grem1*** ^Δ/Δ^; ***Bmp7*** ^Δ/Δ^	**8 (89%)** +	**1 (11%)** +	**0**	**9**
*Grem1* ^Δ/+^; *Bmp7* ^Δ/Δ^	15 (94%)+	1 (6%)+	0	16
*Bmp7* ^Δ/Δ^	6 (100%)+	0	0	6

Shown are the numbers of newborn mice with two, one or no kidneys.

*Hypodysplastic kidneys.

+ All kidneys of *Bmp7*-deficient mice are hypodysplastic due to the essential *Bmp7* requirement for nephrogenesis.

**Figure 3:**
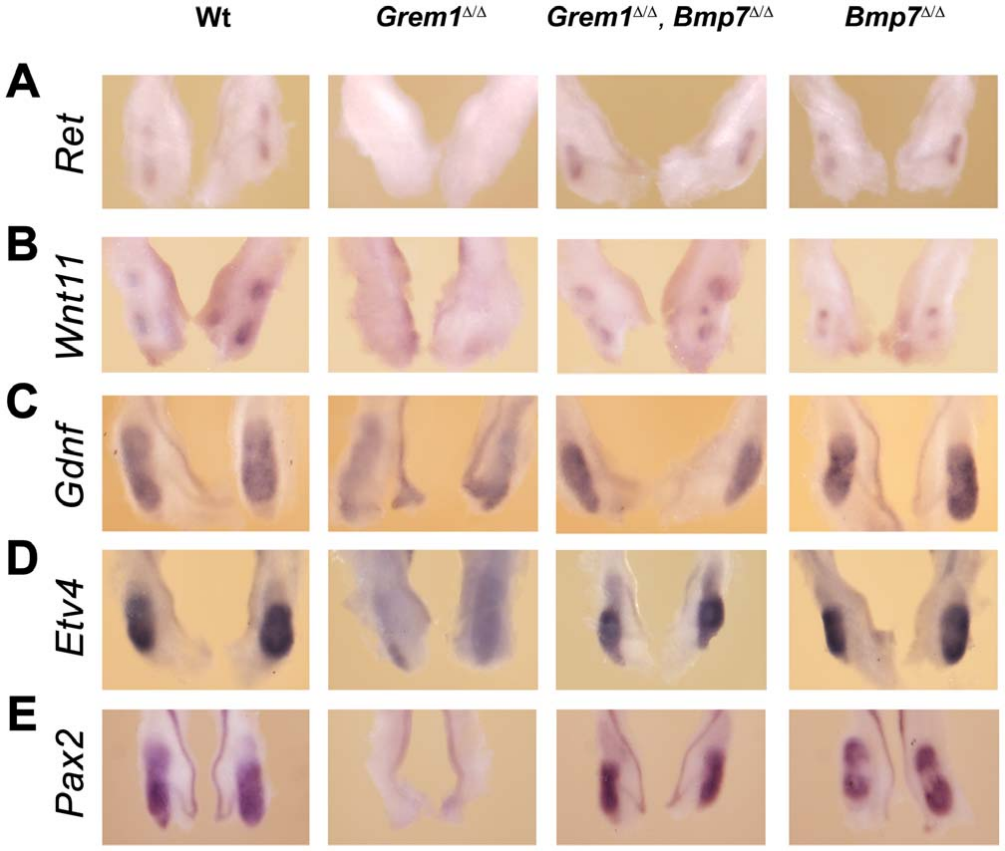
Restoration of ureteric bud outgrowth, branching and GNDF/Wnt11 feedback signaling in *Grem1* ^Δ/Δ^, *Bmp7* ^Δ/Δ^ embryos. Comparative *in situ* hybridization analysis of *Ret* (A), *Wnt11* (B), *Gdnf* (C), *Etv4* (D) and *Pax2* (E) expression in wild-type (Wt; +/+), *Grem1*-deficient, *Grem1* ^Δ/Δ^, *Bmp7* ^Δ/Δ^ and *Bmp7* ^Δ/Δ^ kidneys at embryonic day E11.5–11.75 (49–54 somites). (A, B) *Ret* and *Wnt11* expression were absent from the arrested ureteric bud in *Grem1* ^Δ/Δ^ embryos (Michos et al. 2004, 2007), but their expression was restored in kidney rudiments of *Grem1* ^Δ/Δ^, *Bmp7* ^Δ/Δ^ embryos. (C, D) In addition, the expression of mesenchymal *Gdnf* and the transcriptional target *Etv4* was propagated in *Grem1* ^Δ/Δ^, *Bmp7* ^Δ/Δ^ embryos in contrast to their rapid loss from *Grem1* ^Δ/Δ^ kidney rudiments. Note that the labeling of the Wolffian duct and ureteric epithelium by the *Gdnf in situ* hybridization probe is non-specific. (E) *Pax2* expression was also restored in the metanephric mesenchyme of *Grem1* ^Δ/Δ^, *Bmp7* ^Δ/Δ^ embryos, while its expression is lost from *Grem1* ^Δ/Δ^ mutants concurrent with massive apoptosis [17].

**Figure 4:**
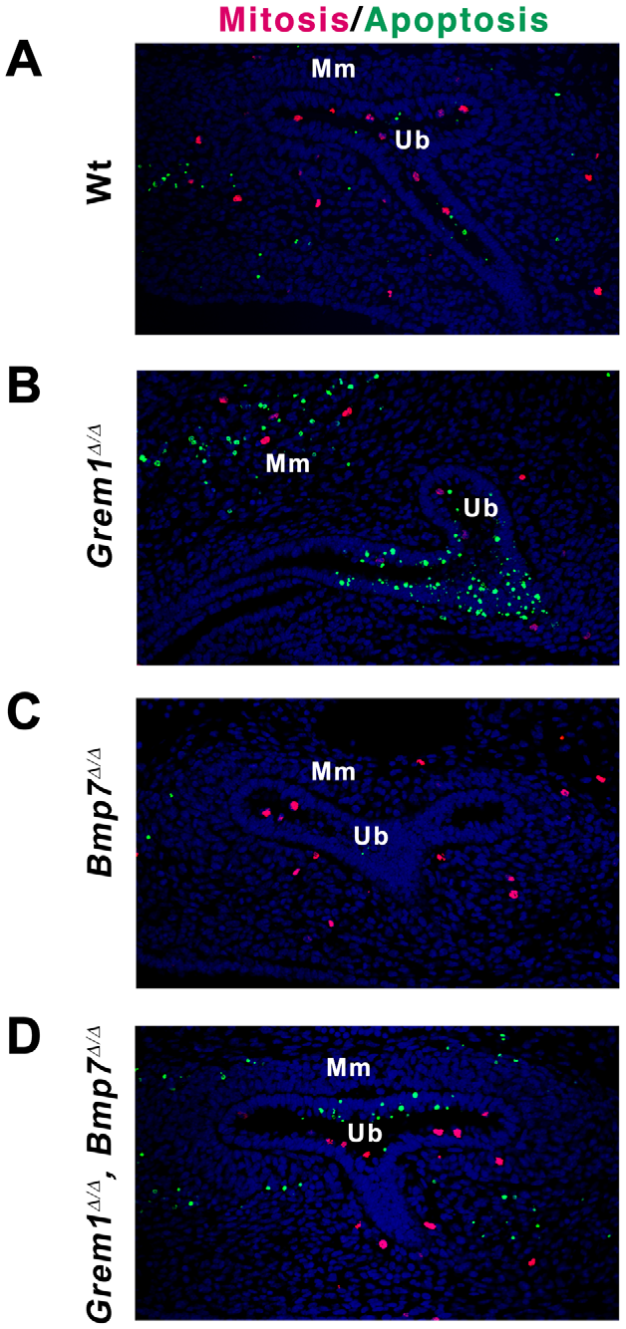
The massive apoptosis in *Grem1*-deficient metanephric kidney rudiments is suppressed by the *Bmp7* inactivation. Apoptotic cells were detected by TUNEL (green fluorescence) on serial histological sections of kidney rudiments at E11.5. Concurrently, mitotic cells were revealed by detection of nuclear phospho-histone H3 proteins (pH 3, red fluorescence) and the overall cell density was assessed by staining nuclear DNA with Hoechst 33258 (blue fluorescence). Representative sections are shown for all genotypes. (A) Wild-type metanephros. Note the condensation of mesenchymal cells (blue nuclei) around the invading ureteric tips. (B) *Grem1*-deficient metanephros. Massive apoptosis was observed in both the mesenchyme and ureteric bud epithelium (green) and mesenchymal cells remained loose (blue). Only few mitotic cells (red) were detected. (C) *Grem1* ^Δ/Δ^, *Bmp7* ^Δ/Δ^ metanephros. Cellular apoptosis in both compartments was almost completely suppressed and proliferation, mesenchymal condensation and branching of the ureteric epithelium were restored. Note that the apoptosis in the distal part of the branching ureteric epithelium remained increased. (D) The development of the *Bmp7*-deficient metanephros was comparable to wild-type controls at this developmental stage. Ub: ureteric bud epithelium; Mm: metanephric mesenchyme.

This morphological analysis indicated that additional inactivation of *Bmp7* in *Grem1*-deficient mouse embryos had restored the morpho-regulatory networks that control the initiation of ureteric bud outgrowth (Figure 3). Indeed, the ureteric bud had invaded the metanephric mesenchyme and branched once by embryonic day E11.5 in kidney rudiments lacking both *Grem1* and *Bmp7* (Figure 3A). In particular, the expression of *Ret* and *Wnt11* at the tips of the branching ureteric bud was restored (Figure 3B, compare to Figure 3A) and *Gdnf* expression up-regulated in the surrounding metanephric mesenchyme in *Grem1* ^Δ/Δ^, *Bmp7* ^Δ/Δ^ embryos (Figure 3C). Furthermore, the expression of *Etv4*, a downstream effector of GNDF/RET signaling [9], [10] was similar to wild-type in *Grem1* ^Δ/Δ^, *Bmp7* ^Δ/Δ^ kidneys, which contrasts with its marked down-regulation in *Grem1* ^Δ/Δ^ embryos (Figure 3D). This analysis revealed that GDNF/WNT11 e-m feedback signaling and the onset of ureteric epithelial branching were restored in *Grem1* ^Δ/Δ^, *Bmp7* ^Δ/Δ^ embryos. In agreement with this result, *Pax2* expression was restored to wild-type levels in *Grem1* ^Δ/Δ^, *Bmp7* ^Δ/Δ^ kidneys at E11.5 (Figure 3E). The disruption of e-m feedback signaling in *Grem1*-deficient embryos caused massive cellular apoptosis of the metanephric mesenchyme and ureteric epithelium (Figure 4B), which results in elimination of the metanephros by around E12.5 (for details see refs. [5], [17]). This massive mesenchymal and epithelial apoptosis was mostly suppressed by additional inactivation of *Bmp7* (Figure 4D), which agrees with a previous observation that high BMP7 levels induce apoptosis of cultured collecting duct epithelial cells [25]. In contrast, no significant alterations in mitotic cells were detected among the four different genotypes analyzed (Figure 4). Subsequently, the number of mitotic cells was reduced in *Grem1* ^Δ/Δ^, *Bmp7* ^Δ/Δ^ and *Bmp7*-deficient kidneys as previously reported for the latter (data not shown and ref. [26]).

**Figure 5:**
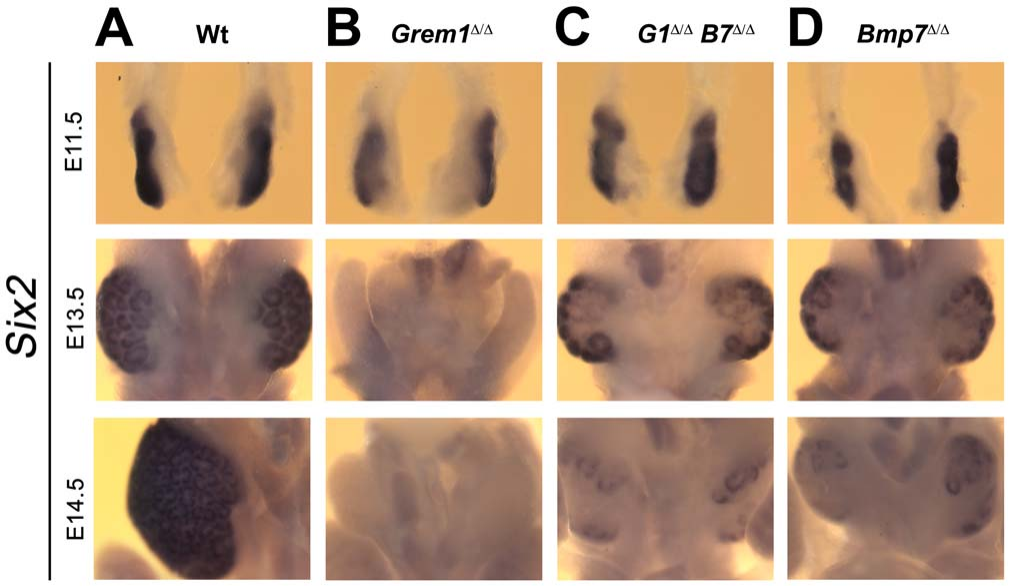
Differential loss of *Six2* expressing progenitors from mutant metanephric kidneys. *Six2* expression was analyzed by whole-mount *in situ* hybridization during initiation (E11.5) and progression of nephrogenesis (E13.5 and E14.5). Representative pairs of developing metanephric kidneys are shown at the same magnifications for the different stages and genotypes to reveal the differences in size. (A) Wild-types. Only one of the two developing kidneys is shown at E14.5 due to size. (B) *Grem1* deficiency. *Six2* expression was lost by E13.5. At E13.5, the developing gonads became visible due to the renal agenesis. (C) *Grem1* ^Δ/Δ^, *Bmp7* ^Δ/Δ^ (*G1* ^Δ/Δ^, *B7* ^Δ/Δ^) metanephric kidneys. *Six2* expression was normal at E11.5, but lost during progression of nephrogenesis (E13.5–E14.5) as in *Bmp7*-deficient kidneys. (D) *Bmp7*-deficient metanephric kidneys.

By E13.5, the formation of glomeruli and collecting ducts was ongoing in *Grem1* ^Δ/Δ^, *Bmp7* ^Δ/Δ^ and *Bmp7*-deficient kidneys (Figure S2), which established that the hypodysplastic phenotypes (Figure 2) were not a consequence of disrupting the onset of nephrogenesis. Therefore, we analyzed the expression of *Six2*, which marks the self-renewing population of nephrogenic progenitors (Figure 5A) [14], [15]. In *Grem1*-deficient embryos, *Six2* expression was completely lost by E13.5 as a consequence of eliminating the metanephros by apoptosis (Figure 5B and Figure 4B) [17]. In contrast, *Six2* expression was normal at E11.5 and *Six2*-expressing progenitors remained at E13.5 in *Grem1* ^Δ/Δ^, *Bmp7* ^Δ/Δ^ and *Bmp7* ^Δ/Δ^ metanephric kidney rudiments (Figure 5C, 5D), which coincided with normal onset of nephrogenesis (Figure S2). However, the lack of *Bmp7* resulted in an almost complete loss of *Six2*-expressing mesenchymal progenitors on both *Bmp7* ^Δ/Δ^ and *Grem1* ^Δ/Δ^, *Bmp7* ^Δ/Δ^ kidneys by E14.5 (Figure 5C, 5D).

This genetic analysis revealed that the normally *Grem1*-dependent initiation of ureteric epithelial outgrowth and branching was restored in *Grem1* ^Δ/Δ^, *Bmp7* ^Δ/Δ^ embryos (Figure 3). In contrast, the *Bmp7*-dependent propagation of *Six2* positive nephrogenic progenitors was not improved (Figure 5), which provides a straightforward explanation for the hypodysplastic kidney phenotype observed in *Grem1* ^Δ/Δ^, *Bmp7* ^Δ/Δ^ embryos (Figure 2).

## Discussion

**Figure 6:**
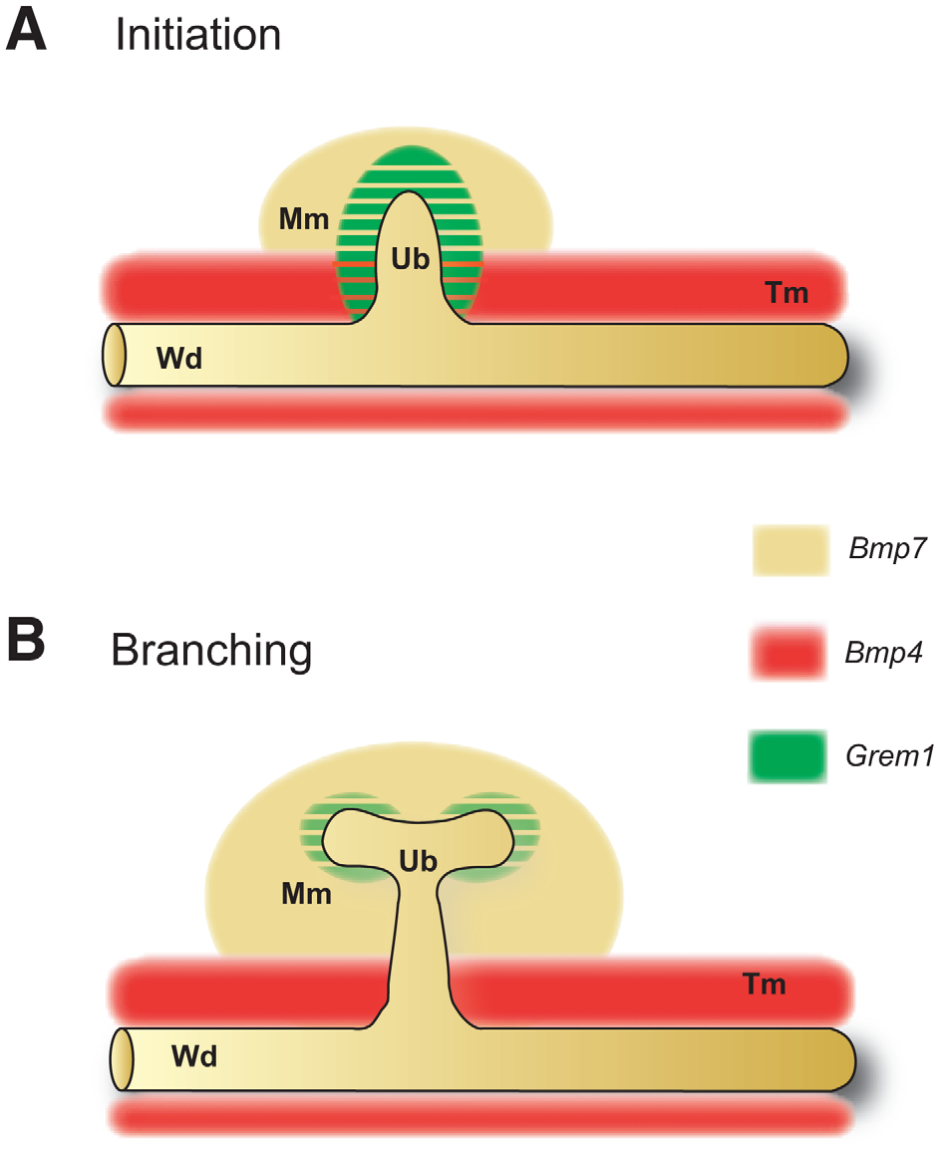
Interactions of GREM1 with BMP7 and BMP4 during ureteric epithelial branching morphogenesis. (A) GREM1-mediated reduction of overall BMP activity enables initiation of ureteric bud outgrowth. (B) GREM1-mediated BMP antagonism during the onset of epithelial branching morphogenesis. *Bmp4* (red) is expressed by the tail bud derived mesenchyme (Tm) enveloping the Wolffian duct (Wd) and ureteric bud epithelia (Ub). *Bmp7* (yellow) is expressed by the nascent mesenchyme (panel A), metanephric mesenchyme (Mm, panel B) and the Wolffian duct and ureteric bud epithelia (Figure 1C). *Grem1* (green) is initially expressed by the mesenchyme surrounding the nascent ureteric bud, where it likely antagonizes both BMP4 and BMP7 activities (panel A). Subsequently, *Grem1* is expressed by the metanephric mesenchyme surrounding the invading ureteric epithelial tips (panel B). The overlap of the *Grem1* with the *Bmp4* (panel A) and *Bmp7* expression domains (panel A, B) is indicated by green stripes.

Our genetic analysis establishes that GREM1 antagonizes BMP7 during the onset of metanephric kidney development, as its genetic inactivation in the context of a *Grem1* deficiency restores ureteric bud outgrowth and epithelial branching. Indeed, cell-biochemical analysis showed that GREM1 binds to BMP7 and regulates its activity in kidney mesangial cells [27]. We established previously that inactivation of one *Bmp4* allele in *Grem1*-deficient mouse embryos also restores ureteric bud outgrowth and branching morphogenesis [5]. *Bmp4* is expressed by the tailbud-derived mesenchyme enveloping the Wolffian duct and nascent ureteric bud [5], [22], while *Bmp7* is expressed by the cap mesenchyme (located at the tip of the ureteric bud) and at lower levels by the entire metanephric mesenchyme and ureteric epithelium (this study and refs. [16], [17]. The fact that *Bmp4* but not *Bmp7* is expressed by the mesenchyme enveloping the Wolffian duct (Figure 6A) provides a likely explanation for the fact that an additional epithelial bud forms in *Bmp4* but not *Bmp7*-deficient kidney rudiments (this study). During this initial phase, *Grem1* is expressed by the mesenchyme that surrounds the nascent ureteric bud (Figure 6A) [5], [17]. GREM1 antagonism of both BMP4 and BMP7 reduces overall BMP activity locally, which enables the ureteric bud to invade the metanephric mesenchyme and initiate branching (Figure 6A, 6B). *Grem1* expression is highly dynamic and is lost from the mesenchyme enveloping the ureter stalk as it becomes expressed by the cap mesenchyme that surrounds the tips of the branching ureter (Figure 6B) [5], [17]. This enables BMP4 to induce differentiation of the ureteric stalk during subsequent development [5], [17] while *Grem1* expression around the ureteric epithelial tips may regulate branching [2], [5]. The restoration of ureteric epithelial branching morphogenesis by either inactivating *Bmp7* or reducing *Bmp4* in the context of the *Grem1* deficiency (this study and ref. [5]) suggests that the major essential function of GREM1 during metanephric kidney development is the overall reduction of BMP activity around the nascent ureteric bud rather than inhibition of a particular BMP ligand (Figure 6A).

Furthermore, it was shown that BMP4 expressed under the control of the *Bmp7* locus compensates for the loss of *Bmp7* during metanephric kidney, but not eye development [20]. To gain further insight into the interactions of the two BMP ligands during kidney development, we attempted to generate mouse embryos lacking *Bmp7* constitutively and *Bmp4* conditionally. This turned out to be impossible, as inactivation of only one *Bmp4* allele in *Bmp7* ^Δ/Δ^ embryos causes embryonic lethality around E9.0 similar to *Bmp4* ^Δ/Δ^, but sharply contrasting with *Bmp7* ^Δ/Δ^ and *Bmp7* ^Δ/+^, *Bmp4* ^Δ/+^ mouse embryos (A.G. and R.Z., unpublished results). This provides further evidence in favor of a strong genetic interaction between these two BMP ligands. One possible explanation could be that they form functional heterodimers that assemble with BMP receptors to initiate signal transduction. In fact this has been suggested, as *Bmp7* ^Δ/+^, *Bmp4* ^Δ/+^ mice display minor skeletal defects [28]. BMP7 also forms heterodimers with BMP4 and BMP2 during patterning of the dorso-ventral body axis in both *Xenopus laevis* and zebrafish embryos [29], [30]. In addition, the mesoderm-inducing potential of BMP4–BMP7 heterodimers was shown to be much stronger than that of respective homodimers [31]. These functions of BMP4–BMP7 heterodimers in dorso-ventral axis formation and gastrulation of vertebrate embryos provide a likely explanation for the observed mid-gestational lethality of *Bmp7* ^Δ/Δ^, *Bmp4* ^Δ/+^ mouse embryos.

In *Grem1* ^Δ/Δ^, *Bmp7* ^Δ/Δ^ kidney rudiments, GDNF/WNT11 e-m feedback signaling [12] was restored in agreement with initially normal ureteric epithelial branching. At early stages, the expression of *Six2* was normal and nephrogenesis initiated in contrast to *Grem1*-deficient kidneys. However, *Grem1* ^Δ/Δ^, *Bmp7* ^Δ/Δ^ embryos succumb to premature depletion of nephrogenic progenitors during further development. Their hypodysplastic phenotype reveals the prevalence of the *Bmp7* kidney phenotype (ref. [26] and this study). Therefore, the spatio-temporally controlled antagonism of BMP activity by GREM1 appears limited to ureteric bud outgrowth and branching. This antagonistic interaction precedes the function of BMP4 in ureter stalk differentiation [21], [22] and BMP7 in expanding the nephrogenic progenitors [26].

It has recently been shown that siRNA-mediated inhibition of *Grem1* expression is beneficial for the diabetic kidney by enabling maintenance of BMP7 activity, which in turn contributes to ameliorating the renal damage [27]. Therefore, aberrant re-activation of the morpho-regulatory GREM1–BMP7 interactions identified in our study may underlie disease initiation and/or progression in diabetic nephropathies.

## Materials and Methods

### Ethics Statement

All genetic studies involving mice were performed in strict accordance with Swiss law following approval by the Joint Commission on Experiments involving Animals of Argovia and both Cantons of Basel (Gemeinsame Tierversuchskommission der Kantone Aargau, Basel-Land und Basel-Stadt). The relevant license no. 1950 entitled “Regulation of mouse limb and kidney organogenesis by interaction of Gremlin1 with BMPs” (valid until 13-12-2011) was issued by the Veterinary Office of Basel. All animal experiments were classified as grade zero, which implies minimal suffering of mice and the 3R principles were strictly implemented as required by the Swiss laws governing experimental studies involving animals.

### Mouse Strains

As the *Grem1* and *Bmp7* loci are both located on the mouse chromosome 2, the following genetic approach was used to generate the mice for analysis. *Grem1* ^Δ/+^ mice [17] were mated with *Bmp7* ^Δ/+^ mice [19] and double heterozygous offspring were crossed with wild-type mice to identify mice carrying both mutations on the same chromosome. As both loci are about 38 cM apart (UCSC Genome Browser), the recombination frequency was reasonably high, *Grem1* ^Δ/Δ^, *Bmp7* ^Δ/Δ^ embryos and newborn mice were most efficiently generated by inter-crossing males carrying both mutations in *cis* with females carrying the mutant alleles either *cis* or *trans*. Single mutant *Grem1* ^Δ/Δ^ and *Bmp7* ^Δ/Δ^ embryos and newborn mice were generated by inter-crossing heterozygous mice. *Grem1* ^Δ/Δ^, *Bmp4* ^Δ/+^ mutant embryos and mice were generated as previously described [24]. All genetic studies were carried out in a predominant 129/SvEv genetic background to assure maximal penetrance of the renal agenesis phenotypes [5]. Tamoxifen-CRE recombinase (using the CAGGCre-ER™ transgene) [32] mediated conditional inactivation of *Bmp4* in mouse embryos carrying both the null (*Bmp4* ^Δ^) and hypomorphic “floxed” allele (*Bmp4* ^loxP-lacZ^) [33] was done as described in ref. [24]. Briefly, females homozygous for the *Bmp4* ^loxP-lacZ^ allele were mated with *Bmp4* ^Δ/+^, *TMCre* ^tg/+^ males. Pregnant females received an intra-peritoneal injection of a mix of tamoxifen (3 mg, Sigma) and Progesterone (1.5 mg, Sigma) at embryonic days E8.75–E9.5. The predominant 129/SvEv genetic background was used for all studies, as no ectopic ureteric buds were observed in *Bmp4* ^Δ/+^ and *Bmp4* ^Δ/f^ embryos in this genetic background. Mice and embryos were genotyped as described [17], [24], [33].

### Molecular and Immunohistochemical Analysis

Embryos were staged by determining their somite numbers up to embryonic day E12.5. Whole-mount and section RNA *in situ* hybridizations were performed as described [6]. For histological and immuno-histochemical analysis, 7–10 µm sagital sections were prepared from embryonic and newborn kidneys fixed in 4% paraformaldehyde at 4°C (overnight) and paraffin-embedded using standard protocols. Histological sections in wax were firmly attached to Superfrost® Plus slides (Thermo Scientific) by drying them 42°C for 24–48 hours and storing them at 4°C until use. After dewaxing, histological sections were either stained with Haematoxylin and Eosin or periodic acid Schiff solutions (Sigma) to reveal the brush border (microvilli) of the distal and proximal tubules. For immunhistochemical analysis, histological sections were dewaxed and treated as required to detect the antigens of choice (see below). Prior to mounting the slides in Mowiol (Calbiochem), all sections were counterstained for 1 min with Hoechst 33258 (5 mg/ml).

### Analysis of Nephrogenesis

Slides with dewaxed sections were boiled at 120°C in 10 mM sodium citrate (Merck) for 90 seconds to render the antigens accessible to detection [34]. Polyclonal goat α-mouse podocalyxin (1∶50, R&D Systems) and monoclonal mouse α-pan cytokeratin antibodies (1∶50, Sigma) were used according to the manufacturer's instructions in combination with the appropriate secondary antibodies. The use of Cy3-conjugated donkey anti-goat IgGs (red, 1∶100, Amersham) and Cy2-conjugated donkey anti-mouse IgGs (green, 1∶200, Amersham) resulted in detection of forming glomeruli in red and collecting ducts in green.

### Proliferation and Apoptosis Assay

Rabbit polyclonal anti-phospho-histone H3 (pH 3, Ser10, 1∶500, Millipore), which marks mitotic cells, was used to detect cells in mitosis in combination with Cy3-conjugated goat-anti-rabbit IgGs (red, 1∶250, Amersham) according to manufacturer's instructions. After phospho-histone H3 detection, the samples were washed with 10 mM Tris-HCl pH 7.5 and apoptotic cells were visualized using the in situ cell death detection kit (green, Roche).

## Supporting Information

Figure S1 **The collecting duct system and glomeruli in wild-type and mutant newborn mice.** The collecting duct system was revealed by cytokeratins (green fluorescence) and the glomeruli by podocalyxin (red fluorescence) on serial sections of newborn kidneys. Left panels show low magnification overviews (10×), middle panels an enlargement of the cortex (20×, arrowheads point to glomeruli), right panels an enlargement of the medulla (20×). Note that the enlargements are either taken from the same or a close-by serial section. (A) Wild-type control. (B) *Grem1* ^Δ/Δ^, *Bmp7* ^Δ/Δ^ kidneys were always much smaller than wild-type (panel A) and *Grem1* ^Δ/Δ^, *Bmp4* ^Δ/+^ kidneys at birth (panel D). In addition, the numbers of fully developed glomeruli (red) were always reduced in kidneys of *Grem1* ^Δ/Δ^, *Bmp7* ^Δ/Δ^ newborn mice. (C) The hypodysplastic phenotype of *Bmp7* ^Δ/Δ^ kidneys. Note the similar reduction of glomeruli (red; compare to panel C). (D) The restoration of kidney development in *Grem1* ^Δ/Δ^, *Bmp4* ^Δ/*+*^ embryos was corroborated by the analysis of the collecting duct system and glomeruli. Cx: cortex; Cys: cyst; Me: medulla; Pa: papilla.
(TIF)

Figure S2 **Nephrogenesis initiates normally during development of** ***Grem1*** **^Δ/Δ^,** ***Bmp7*** **^Δ/Δ^ metanephric kidneys.** Nephrogenesis was assessed by the distribution of cytokeratins, which mark the forming collecting duct system (green fluorescence) and podocalyxin, which marks nascent glomeruli (red fluorescence) at E13.5. The overall morphology was assessed by counterstaining cell nuclei with Hoechst 33258 (blue). The kidneys of *Grem1* ^Δ/Δ^, *Bmp7* ^Δ/Δ^ embryos were compared to age-matched wild-type and *Bmp7*-deficient counterparts, as in *Grem1*-deficient embryos, the kidney is eliminated already prior to this stage. Analysis of serial sections revealed that the extent of nephrogenesis in *Grem1* ^Δ/Δ^, *Bmp7* ^Δ/Δ^ metanephric kidney rudiments (panel B) was similar to wild-type (panel A) and *Bmp7*-deficient kidneys (panel C) at this developmental stage. Representative illustrations are shown for all three genotypes. The left panels show low magnification overviews, the brackets indicate the high magnification views shown in the right panels. (A) Wild-type metanephros; (B) *Grem1* ^Δ/Δ^, *Bmp7* ^Δ/Δ^ metanephros; (C) *Bmp7* ^Δ/Δ^ metanephros. Cd: collecting ducts; Gl: glomeruli; Mm: metanephric mesenchyme.
(TIF)
