# Supplementary figures and images for: Genetic Analysis Reveals an Unexpected Role of BMP7 in Initiation of Ureteric Bud Outgrowth in Mouse Embryos
Source: PLoS One. 2011 Apr 28;6(4):e19370. doi: 10.1371/journal.pone.0019370 (PMC3084290; doi:10.1371/journal.pone.0019370)

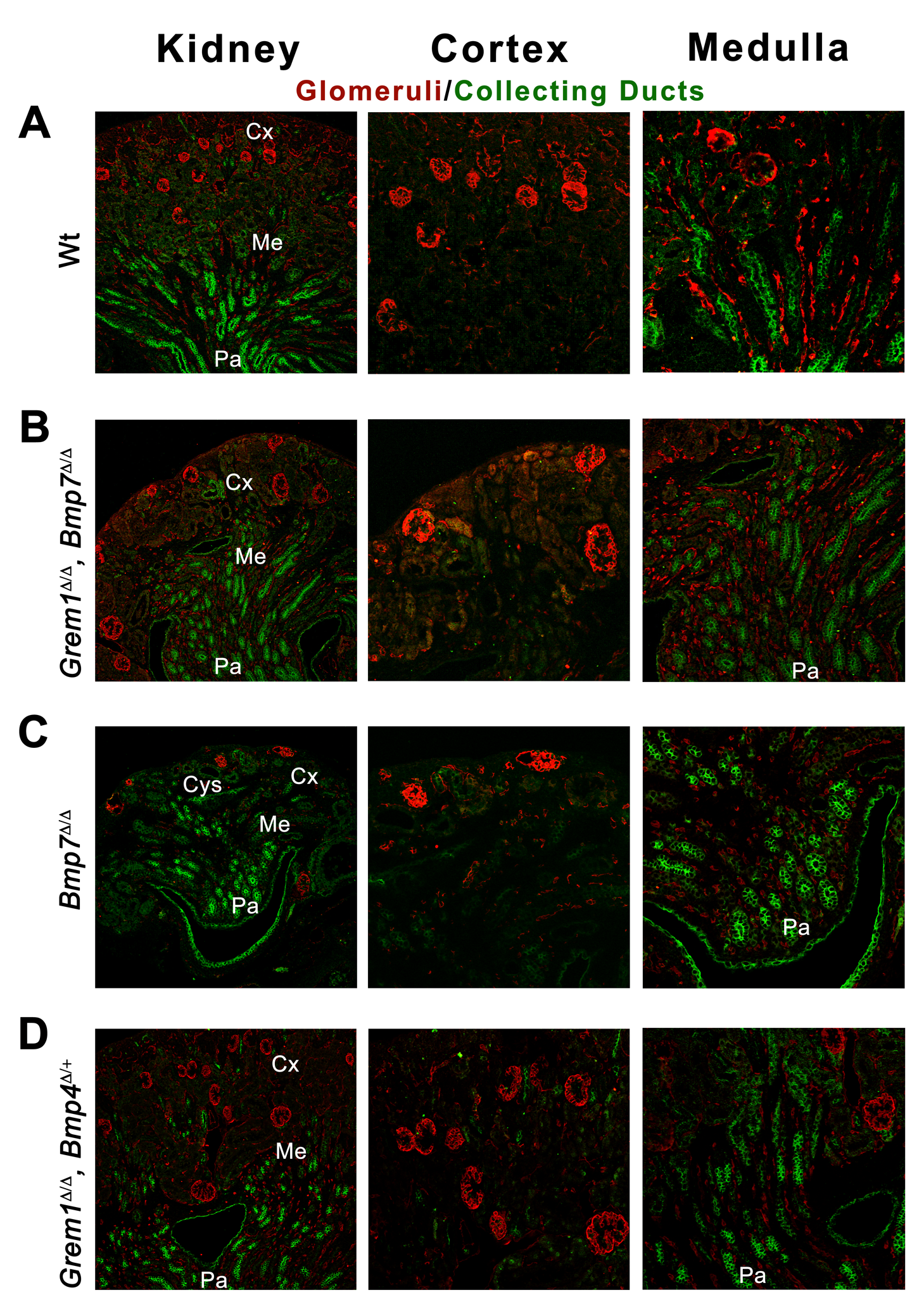

Supplement: Figure S1 — The collecting duct system and glomeruli in wild-type and mutant newborn mice. The collecting duct system was revealed by cytokeratins (green fluorescence) and the glomeruli by podocalyxin (red fluorescence) on serial sections of newborn kidneys. Left panels show low magnification overviews (10×), middle panels an enlargement of the cortex (20×, arrowheads point to glomeruli), right panels an enlargement of the medulla (20×). Note that the enlargements are either taken from the same or a close-by serial section. (A) Wild-type control. (B) Grem1 Δ/Δ, Bmp7 Δ/Δ kidneys were always much smaller than wild-type (panel A) and Grem1 Δ/Δ, Bmp4 Δ/+ kidneys at birth (panel D). In addition, the numbers of fully developed glomeruli (red) were always reduced in kidneys of Grem1 Δ/Δ, Bmp7 Δ/Δ newborn mice. (C) The hypodysplastic phenotype of Bmp7 Δ/Δ kidneys. Note the similar reduction of glomeruli (red; compare to panel C). (D) The restoration of kidney development in Grem1 Δ/Δ, Bmp4 Δ/+ embryos was corroborated by the analysis of the collecting duct system and glomeruli. Cx: cortex; Cys: cyst; Me: medulla; Pa: papilla. (TIF) [file pone.0019370.s001.tif]

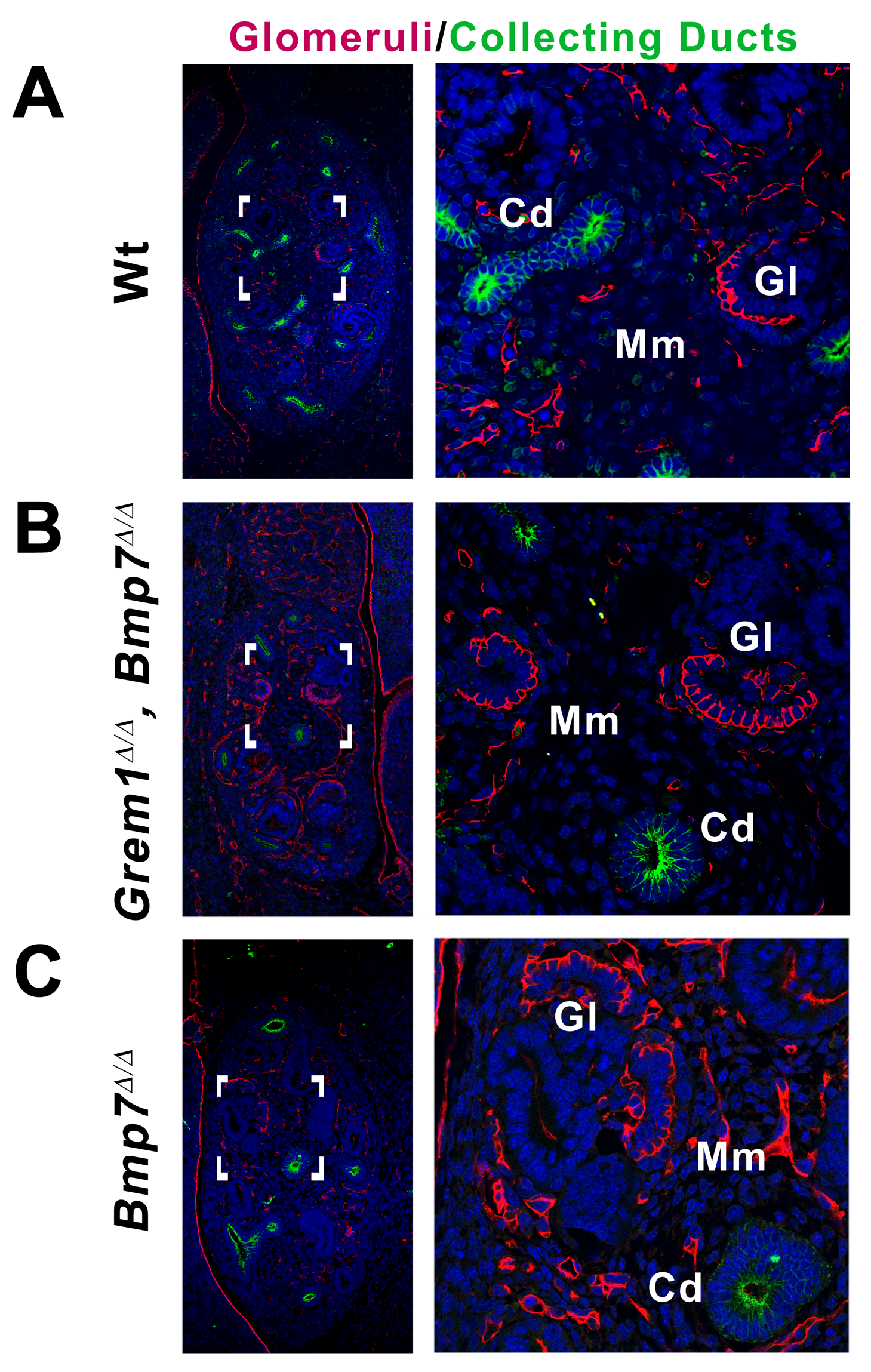

Supplement: Figure S2 — Nephrogenesis initiates normally during development of Grem1 Δ/Δ, Bmp7 Δ/Δ metanephric kidneys. Nephrogenesis was assessed by the distribution of cytokeratins, which mark the forming collecting duct system (green fluorescence) and podocalyxin, which marks nascent glomeruli (red fluorescence) at E13.5. The overall morphology was assessed by counterstaining cell nuclei with Hoechst 33258 (blue). The kidneys of Grem1 Δ/Δ, Bmp7 Δ/Δ embryos were compared to age-matched wild-type and Bmp7-deficient counterparts, as in Grem1-deficient embryos, the kidney is eliminated already prior to this stage. Analysis of serial sections revealed that the extent of nephrogenesis in Grem1 Δ/Δ, Bmp7 Δ/Δ metanephric kidney rudiments (panel B) was similar to wild-type (panel A) and Bmp7-deficient kidneys (panel C) at this developmental stage. Representative illustrations are shown for all three genotypes. The left panels show low magnification overviews, the brackets indicate the high magnification views shown in the right panels. (A) Wild-type metanephros; (B) Grem1 Δ/Δ, Bmp7 Δ/Δ metanephros; (C) Bmp7 Δ/Δ metanephros. Cd: collecting ducts; Gl: glomeruli; Mm: metanephric mesenchyme. (TIF) [file pone.0019370.s002.tif]
